# Predicting the individual probability of macular hole closure following intravitreal ocriplasmin injections for vitreomacular traction release using baseline characteristics

**DOI:** 10.1038/s41598-021-03509-z

**Published:** 2021-12-16

**Authors:** Thomas Bertelmann, Lars Berndzen, Thomas Raber, Sebastian Pfeiffer, Andreas Leha, Christoph Paul, Nicolas Feltgen, Sebastian Bemme

**Affiliations:** 1 Department of Ophthalmology, University Medical Center Goettingen, Robert-Koch-Straße 40, 37075 Goettingen, Germany; 2 Oxurion NV, Leuven, Belgium; 3 UMG Study Center, University Medical Center Goettingen, Goettingen, Germany; 4 Department of Medical Biometry and Statistical Bioinformatics, University Medical Center Goettingen, Goettingen, Germany; 5 Department of Ophthalmology, Philipps-University Marburg, Marburg, Germany

**Keywords:** Retinal diseases, Vision disorders, Vitreous detachment

## Abstract

The primary objective was to create and establish a new formula that predicts the individual probability of macular hole closure for eyes with full thickness macular holes (FTMH) accompanied by vitreomacular traction (VMT) which received enzymatic vitreolysis using intravitreally administered ocriplasmin. The secondary objective was to evaluate the forecast reliability of a previously published formula for VMT resolution in VMT-only eyes (Odds_IVO-Success_ = e^Intercept ^× OR^years ^× OR^ln(µm)^; Probability_IVO-Success_ = Odds_IVO-Success_/(Odds_IVO-Success_ + 1)) on VMT resolution using the current dataset of eyes with FTMH accompanied by VMT. Retrospective analysis of the OASIS, ORBIT, and INJECT-studies. Patients with FTMH and VMT with complete information (n = 213) were included. The effect of gender, age, FTMH diameter, lens status and the presence of epiretinal membranes (ERM) on FTMH closure was assessed using separate univariate logistic regression analyses. With regard to VMT release separate univariate regression analyses were carried out and results were compared with formerly published data of VMT resolution in eyes with VMT only. Overall, 126 eyes (63%) experienced VMT resolution within 28 days. Younger age (p < 0.0001) and VMT diameter (p = 0.041) had a significant impact on VMT release. Overall, 81 eyes (38%) treated with ocriplasmin showed FTMH closure within 28 days. Univariate analysis of the different predictors analyzed revealed that FTMH diameter < 250 µm had a significant impact on treatment success (p = 0.0495). It was not possible to calculate and establish a new multivariate formula that can predict the individual FTMH closure probability for eyes with FTMHs and VMT. However, the results of VMT release prediction in eyes with FTMHs accompanied by VMT matched the prediction of VMT release in eyes with VMT only when using the previously published formula. All in all, predictors for calculating the individual probability of VMT resolution on the one hand and FTMH closure on the other hand are different suggesting diverse pathophysiological mechanisms.

## Introduction

Vitreomacular traction (VMT) can result in full thickness macular hole (FTMH) development, which in turn might cause severe visual loss^1^. Until the approval of ocriplasmin (Jetrea, Oxurion formerly ThromboGenics, Leuven, Belgium) in the United States by the US Food and Drug Administration in 2012 as well as in Europe by the European Medicines Agency in 2013, pars plana vitrectomy was the sole option as a therapeutic approach^2^. Nowadays, the ophthalmologist’s armamentarium is somewhat broader, still including pars plana vitrectomy, but also intravitreal ocriplasmin or gas injections.

Regarding the use of ocriplasmin, the initial MIVI trials (TG-MV-006 and TG-MV-007) showed that 28 days after a single intravitreal injection 40.6% had a non-surgical FTMH closure as compared to 10.6% of placebo treated eyes (p < 0.001)^3^. As rates of treatment success vary substantially between 12 and 50% in the literature, the key for treatment success seems to be valid patient selection^4^. Variability was also evident in eyes with VMT only scheduled for enzymatic vitreolysis. Here, the rates of successful VMT releases varied between 26.5 and 71%^3,5^. Recently our group published a multivariate formula to calculate the individual probability for VMT release and we were able to show that predicted success rates matched in-vivo treatment results^6^. Such a formula that predicts treatment success on an individual approach is of substantial benefit for patients and ophthalmologists involved when discussing next treatment steps. Patients with a good probability for cleavage of the posterior hyaloid from the internal limiting membrane (ILM) could be routed to enzymatic vitreolysis, whereas patients with a poorer chance might be scheduled for pars plana vitrectomy.

The project described herein aims to establish a formula to estimate the individual FTMH closure probability, and thus to support patients and ophthalmologists in their decision whether or not to treat a FTMH accompanied by VMT with ocriplasmin. As a secondary endpoint further analyses were performed to evaluate, if the forecast reliability of a previously published formula that can predict VMT release in VMT eyes only^6^ might be transferred and applied for eyes with FTMHs and VMT in regard to VMT resolution as well.

## Methods

We retrospectively analyzed an excerpt from patient data retrieved from the prospective phase III and IV-studies OASIS, ORBIT^7^ and INJECT^8^. To be included into our analysis, the dataset of each patient needed the following information at minimum: gender, age, FTMH diameter at baseline, lens status, epiretinal membrane (ERM) visible on optical coherence tomography (OCT) at baseline, VMT-size at baseline, and FTMH closure as well as VMT resolution within 28 days after ocriplasmin injection.

In the OASIS and ORBIT studies FTMH diameter was assessed by a Central Reading Center (CRC) and measured as the minimal linear diameter. In the INJECT study no CRC was involved and FTMH measurements were performed “according to the local practice of the investigator”. We assume that FTMH measurements were performed accordingly as the measurement of the minimal linear diameter was the standard of care at the time the studies were conducted^9^. To analyze FTMH diameter both, radial star as well as line scans, were permitted.

246 patients out of overall 1133 participants in the OASIS (n = 146 patients (8)), ORBIT (n = 539 patients (9)) and INJECT (n = 448 patients (10)) studies with VMT accompanied by a FTMH were identified. Out of this group of interest patients with any missing value (n = 33) were excluded from further evaluation. OCT-related parameters were assessed by an independent and masked central reading center in OASIS and ORBIT as well as by the investigators in INJECT.

In the present post-hoc evaluation, statistical analysis was done using R (V3.6.2, open source) at a significance level of 5%. Categorial data were presented using absolute and relative frequencies. For continuous data, the mean and standard deviation (SD) are presented along with minimum and maximum values (mean ± SD (min–max)).

Univariate logistic regression analyses with pairwise contrast tests were used to compare the single parameters between the study groups as well as to analyze the effect of single parameters on treatment success, which we defined as FTMH closure as well as VMT release as visible on OCT. In regard to FTMH closure, no adjustment for multiple testing was performed due to the explorative approach. A multivariate logistic regression analysis was not conducted for FTMH closure due to only one significant predictor in the univariate analysis. A multivariate analysis was also not performed for VMT release as this was shown in our previous publication^6^. In addition to p-values for the influence as predictors for treatment success, Odds Ratios (ORs) were calculated to estimate the strength of influence, each with a 95% confidential interval (CI). For the analysis of individual VMT release as well as FTMH closure probabilities in eyes with VMT and FTMH, a previously published formula (Model A, including age and VMT diameter: (Odds_IVO-Success_ = e^Intercept ^× OR^years ^× OR^ln(µm)^; Probability_IVO-Success_ = Odds_IVO-Success_/(Odds_IVO-Success_ + 1)) was used and the creation and establishment of the latter were recently described^6^. Model A includes the calculated intercept, age (years) and the horizontal VMT diameter (ln) in µm. In model B gender was also included but provided no additional information and accuracy so that Model A was prefered and chosen. Here, the individual probability of success was calculated for every patient. Patients with similar predicted success probabilities were grouped in 20% intervals and average de facto success rates were calculated for these groups.

Prior to start, the study protocol and the analysis plan were submitted and approved by the local institutional review board (No. 28/1/19, dated 31/Jan/2019; Ethics Committee of University Medical Center Goettingen, Georg-August University Goettingen, Germany). Written informed consent was obtained from all patients for trial participation as well as subsequent secondary analyses of the data prior to study start. All methods were carried out in accordance with local, national, and international guidelines and regulations.

## Results

### Study population

Overall, 213 patients diagnosed with a FTMH accompanied by VMT were included into this analysis (OASIS 44 (20.7%), ORBIT 112 (52.6%), and INJECT 57 (26.8%)). Basic patient characteristics for the complete dataset as well as for each study analyzed are shown in Table 1.

**Table 1:** Basic patient characteristics.

	Complete dataset n = 213	OASIS n = 44 (20.7%)	ORBIT n = 112 (52.6%)	INJECT n = 57 (26.8%)
Gender (female)	164 (77%)	34 (77.3%)	87 (77.7%)	43 (75.4%)
Age (years) Mean ± standard deviation (min–max)	67.3 ± 7.7 (45–89)	66.5 ± 6.3 (49–79)	66.6 ± 7.4 (45–88)	69.2 ± 9.1 (46–89)
Age < 65 years of age	77 (36.2%)	17 (38.6%)	42 (37.5%)	18 (31.6%)
FTMH diameter (µm) mean ± standard deviation (min–max)	282 ± 148 (75–1121)	309 ± 189 (75–1121)	273 ± 147 (75–810)	278 ± 111 (80–663)
FTMH diameter (µm) < 250 µm	105 (49.3%)	19 (43.2%)	62 (55.4%)	24 (42.1%)
FTMH diameter (µm) ≤ 400 µm	180 (84.5%)	34 (77.3%)	92 (82.1%)	54 (94.7%)
VMT diameter (µm) mean ± standard deviation (min–max)	357 ± 366 (10–4211)	464 ± 635 (36–4211)	348 ± 263 (31–1755)	290 ± 214 (10–1212)
Lens status (phakic)	184 (86.4%)	38 (86.4%)	89 (79.5%)	57 (100%)
ERM formation not evident	194 (91.1%)	39 (88.6%)	99 (88.4%)	56 (98.2%)

For almost all parameters analyzed there were no significant differences between the studies (all p > 0.05; data not shown). In the INJECT study a significantly higher number of patients were phakic in comparison to both other studies (p = 0.0473 (versus OASIS) and p = 0.0189 (versus ORBIT).

### FTMH closure

Overall, 81 eyes (38%) treated with ocriplasmin showed FTMH closure within 28 days. FTMH closed in 59.1% (26 eyes) in the OASIS study, 28.6% (32 eyes) in the ORBIT study, and 40.4% (23 eyes) in the INJECT study. In the OASIS study significantly more successful treatments were observed as compared to the ORBIT study (p = 0.0006). All other comparisons between studies showed no significant differences. The univariate analysis of the different predictors analyzed is shown in Table 2. Only FTMH diameter < 250 µm (p = 0.0495) had a significant impact on FTMH closure rates. Other parameters did not have a significant impact (each p > 0.05).

**Table 2:** Univariate analysis of the different predictors for FTMH closure.

Predictor	Total n = 213 (100%)	FTMH closed n = 81 (38%)	FTMH open n = 132 (62%)	Overall p-value	Odds ratio	Overall	INJECT	OASIS	ORBIT
**Gender**									
Female	n = 164 (77%)	n = 64 (79%; 39%)	n = 100 (75.8%; 61%)	0.5840	Female vs male	1.205 (0.625–2.380)	0.872 (0.257–3.07e + 00)	1.615 (0.381–6.898	1.350 (0.505–4.051)
Male	n = 49 (23%)	n = 17 (21%; 34.7%)	n = 32 (24.2%; 65.3%)						
**Age**									
Years Mean ± SD	67 ± 7.7	67 ± 7.7	67 ± 7.7	0.6612	Each year	0.992 (0.957–1.030)	1.024 (0.965–1.09e + 00)	0.886 (0.783–0.985)	0.994 (0.939–1.052)
< 65 years Mean ± SD	n = 77 (36.2%)	n = 32 (39.5%; 41.6%)	n = 45 (34.1%; 58.4%)	0.4249	< 65 years vs ≥ 65 years	1.263 (0.710–2.240)	1.280 (0.406–3.99e + 00)	2.229 (0.635–8.642)	1.000 (0.421–2.314)
≥ 65 years Mean ± SD	n = 136 (63.8%)	n = 49 (60.5%; 36%)	n = 87 (65.9%; 64%)						
**FTMH diameter**									
Mean ± SD (range [µm])	282 ± 148 (75–1121)	262 ± 157 (80–1121)	294 ± 142 (75–810)	0.1259	Each µm	0.998 (0.996–1.000)	0.996 (0.990–1.00e + 00)	1.002 (0.999–1.007)	0.994 (0.990–0.998)
< 250 µm	n = 105 (49.3%)	n = 47 (58%; 44.8%)	n = 58 (43.9%; 55.2%)	0.0468	< 250 µm vs ≥ 250 µm	1.764 (1.011–3.100)	2.718 (0.924–8.35e + 00)	0.917 (0.271–3.125)	2.687 (1.136–6.795)
≥ 250 µm	n = 108 (50.7%)	n = 34 (42%; 31.5%)	n = 74 (56.1%; 68.5%)						
< 400 µm	n = 180 (84.5%)	n = 71 (87.7%; 39.9%)	n = 109 (82.6%; 60.1%)	0.3222	< 400 µm vs ≥ 400 µm	1.498 (0.689–3.470)	1.375 (0.124–3.07e + 01)	0.543 (0.103–2.324)	4.355 (1.154–28.522)
≥ 400 µm	n = 33 (15.5%)	n = 10 (12.3%; 28.6%)	n = 23 (17.4%; 71.4%)						
**Lens status**									
Phakic	184 (86.4%)	n = 70 (86.4%; 38%)	n = 114 (86.4%; 62%)	0.9908	Phakic vs pseudophakic	1.005 (0.454–2.310)	NA	0.688 (0.088–3.983)	0.893 (0.337–2.558)
Pseudophakic	29 (13.6%)	n = 11 (13.6%; 37.9%)	n = 18 (13.6%; 62.1%)						
**ERM formation**									
Absent	194 (91.1%)	n = 75 (92.6%; 38.7%)	n = 119 (90.2%; 61.3%)	0.5453	ERM absent vs ERM present	1.366 (0.516–4.030)	0.000 (NA–6.87e + 121)	2.400 (0.358–19.877)	1.381 (0.389–6.487)
Present	19 (8.9%)	n = 6 (7.4%; 31.6%)	n = 13 (9.8%; 68.4%)						

The third column lists total number of patients with the characteristics of each parameter indicated by the first and second column, and the rate displayed in brackets in column 3 refers to a total of 213 patients. The fourth and fifth columns contain number of patients with closed or opened FTMH: the first rate refers to the total number of patients with FTMH closed (n = 81) and FTMH open (n = 132), respectively, and the second rate refers to the total number of patients with the same characteristic displayed in the third column within the same row.

### VMT resolution

Overall, 126 eyes (59.2%) treated with ocriplasmin showed VMT resolution within 28 days. VMT resolution was achieved in 59.1% (26 eyes) in the OASIS study, 62.5% (70 eyes) in the ORBIT study, and 52.6% (30 eyes) in the INJECT study. The univariate analysis of the different predictors analyzed is shown in Table 3. Overall age (p < 0.0001), age younger than 65 years (p = 0.0067) and VMT diameter (p = 0.041) had a significant impact on VMT release.

**Table 3:** Univariate analysis of the different predictors for VMT release.

Predictor	Total n = 213 (100%)	VMT released n = 126 (63%)	VMT persistent n = 87 (37%)	Overall p-value	Odds ratio	Overall	INJECT	OASIS	ORBIT
**Gender**									
Female	164 (77%)	98 (77.8%)	66 (75.9%)	0.7441	Female vs male	1.114 (0.579–2.120)	1.684 (0.501–5.924)	1.615 (0.381–6.898)	0.734 (0.273–1.843)
Male	49 (23%)	28 (22.2%)	21 (24.1%)						
**Age**									
Years Mean ± SD	67 ± 7.7	65 ± 6.9	70 ± 7.8	< 0.0001	Each year	0.905 (0.865–0.943)	0.933 (0.868–0.994)	0.886 (0.783–0.985)	0.890 (0.830–0.946)
< 65 years Mean ± SD	77 (36.2%)	55 (43.7%)	22 (25.3%)	0.0067	< 65 years vs ≥ 65 years	2.289 (1.271–4.220)	2.229 (0.635–8.642)	2.229 (0.635–8.642)	2.695 (1.178–6.550)
≥ 65 years Mean ± SD	136 (63.8%)	71 (56.3%)	65 (74.7%)						
**FTMH diameter**									
Mean ± SD (range [µm])	282 ± 148 (75–1121)	286 ± 163 (80–1121)	276 ± 124 (75–734)	0.6409	Each µm	1.000 (0.999–1.002)	1.001 (0.996–1.006)	1.002 (0.999–1.007)	0.999 (0.997–1.002)
< 250 µm	105 (49.3%)	63 (50%)	42 (48.3%)	0.8046	< 250 µm vs ≥ 250 µm	1.071 (0.620–1.854)	0.623 (0.213–1.791)	0.917 (0.271–3.125)	1.414 (0.655–3.068)
≥ 250 µm	108 (50.7%)	63 (50%)	45 (51.7%)						
< 400 µm	180 (84.5%)	107 (84.9%)	73 (83.9%)	0.8409	< 400 µm vs ≥ 400 µm	1.080 (0.501–2.280)	0.538 (0.024–5.945)	0.543 (0.103–2.324)	1.875 (0.700–5.037)
≥ 400 µm	33 (15.5%)	19 (15.1%)	14 (16.1%)						
**Lens status**									
Phakic	184 (86.4%)	111 (88.1%)	73 (83.9%)	0.3827	Phakic vs pseudophakic	1.419 (0.641–3.128)	NA	0.688 (0.088–3.983)	2.145 (0.846–5.509)
Pseudophakic	29 (13.6%)	15 (11.9%)	14 (16.1%)						
**ERM formation**									
Absent	194 (91.1%)	117 (92.9%)	77 (88.5%)	0.2776	ERM absent vs ERM present	1.688 (0.652–4.434)	6,643,630.233 (0.000–NA)	2.400 (0.358–19.877)	1.500 (0.451–4.855)
Present	19 (8.9%)	9 (7.1%)	10 (11.5%)						
**VMT diameter**									
Mean ± SD (range [µm])	357 ± 366 (10–4211)	310 ± 250 (10–1755)	424 ± 480 (10–4211)	0.0414	Each µm	0.999 (0.998–1.000)	0.997 (0.994–1.000)	0.999 (0.997–1.000)	0.999 (0.997–1.000)

The third column lists total number of patients with the characteristics of each parameter indicated by the first and second column, and the rate displayed in brackets in column 3 refers to a total of 213 patients. The fourth and fifth columns contain number of patients with released or persistent VMT: the first rate refers to the total number of patients with VMT release (n = 126) and VMT persistence (n = 87), respectively, and the second rate refers to the total number of patients with the same characteristic displayed in the third column within the same row.

### FTMH closure and VMT release

Overall, 67 eyes (31.5%) treated with ocriplasmin showed both, VMT release and FTMH closure within 28 days. In the OASIS, ORBIT, and INJECT studies 26 eyes (59.1%), 27 eyes (24.1%), and 14 eyes (24.6%) had a complete posterior vitreous detachment (PVD) and a closed FTMH 28 days after the injection. The univariate analysis of the different predictors analyzed is shown in Table 4. Only age (p < 0.0001) and age younger than 65 years (p = 0.0497) had a significant impact.

**Table 4:** Univariate analysis of the different predictors for VMT release.

Predictor	Total n = 213 (100%)	FTMH closed + VMT released n = 67 (31.5%)	All other results n = 146 (68.5%)	Overall p-value
**Gender**				
Female	164 (77%)	54 (80.6%)	110 (75.3%)	0.7801
Male	49 (23%)	13 (19.4%)	36 (24.7%)	
**Age**				
Years mean ± SD	67 ± 7.7	65.8 ± 6.5	67.9 ± 8.2	< 0.0001
< 65 years mean ± SD	77 (36.2%)	28 (41.8%)	49 (33.6%)	0.0479
≥ 65 years mean ± SD	136 (63.8%)	39 (58.2%)	97 (66.4%)	
**FTMH diameter**				
Mean ± SD (range [µm])	282 ± 148 (75–1121)	265 ± 166	268.3 ± 127.6	0.2945
< 250 µm	105 (49.3%)	39 (58.2%)	66 (45.2%)	0.2161
≥ 250 µm	108 (50.7%)	28 (41.8%)	80 (54.8%)	
< 400 µm	180 (84.5%)	58 (86.6%)	120 (82.2%)	0.6627
≥ 400 µm	33 (15.5%)	9 (13.4%)	26 (17.8%)	
**Lens status**				
Phakic	184 (86.4%)	57 (85.1)	127 (86.9%)	0.4325
Pseudophakic	29 (13.6%)	10 (14.9%)	19 (13.1%)	
**ERM formation**				
Absent	194 (91.1%)	63 (94%)	127 (87%)	0.5867
Present	19 (8.9%)	4 (6%)	19 (13%)	
**VMT diameter**				
Mean ± SD (range [µm])	357 ± 366 (10–4211)	329.9 ± 285.5		0.0826

The third column lists total number of patients with the characteristics of each parameter indicated by the first and second column, and the rate displayed in brackets in column 3 refers to a total of 213 patients. The fourth and fifth columns contain number of patients with FTMH closure and VMT release as compared to all other results.

**Figure 1:**
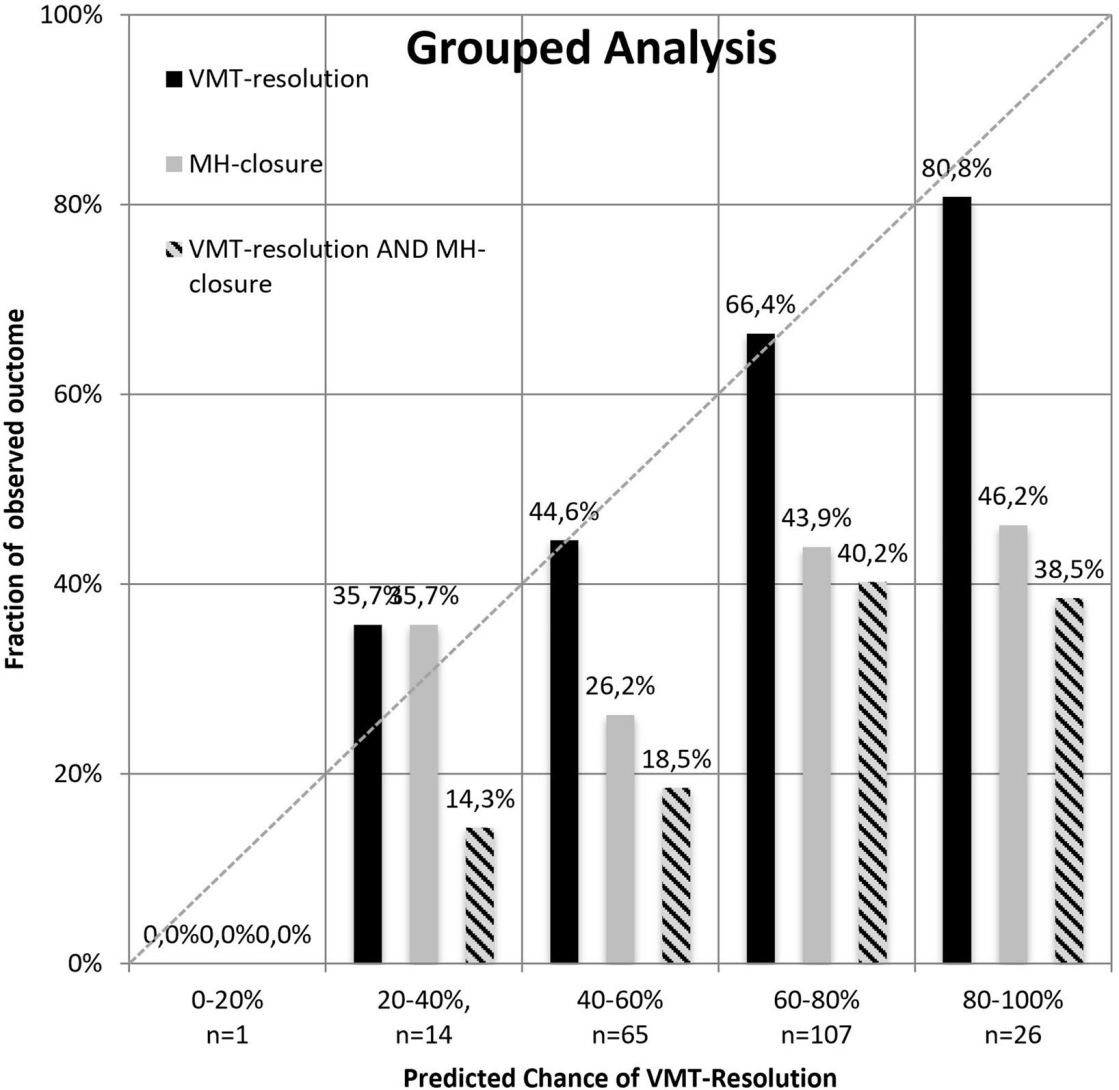
Predicted success rates for VMT release (black), FTMH closure (grey)), as well as FTMH closure in combination with VMT release (striped) as grouped in 20% intervals (x-axis) and average de facto observed success rates (y-axis).

Figure 1 shows predicted success rates for FTMH closure, VMT release, and the combination of FMTH closure and VMT release as grouped in 20% intervals and average de facto observed success rates.

For both conditions, FTMH closure as well as FTMH closure in combination with VMT release, we were not able to calculate a new multivariate prediction model with the current data set.

## Discussion

### Prediction of FTMH closure

In our recent publication of VMT release in pure VMT, many predictors, including gender, age, lens status, ERM formation and VMT diameter had a significant impact on VMT resolution in a univariate analysis, which led to the stratification of these factors and creation of a multivariate formula^6^. Furthermore, the subsequent analysis of the initial MIVI trials (TG-MV-006 and TG-MV-007) by Haller et al. showed that an age from 65 to 75 years, and thus older patients, female gender, ERM formation as well as a pseudophakic lens status had an impact on FTMH closure, beside others^10^. Therefore we aimed to analyze those factors and to create a new multivariate formula to predict FTMH closure after intravitreally administered ocriplasmin, which in turn is the novelty of this project. In the current study however only FTMH diameter was found to be a predictive parameter linked to FTMH closure (Table 2). Although being an explorative approach herein, this observation is in line with a recently published analysis by Joondeph et al. who also showed that FTMH size is the only significant predictor for FTMH closure following intravitreal ocriplasmin use^11^. Consequently, the development of a multivariate model to predict an individualized probability for FTMH closure in eyes with FTMHs using intravitreally administered ocriplasmin was not possible with the datasets of the OASIS, ORBIT and INJECT studies. The data show that all these variables, except FTMH diameter itself, seem to have only a limited impact on FTMH-closure in eyes with FTMHs. This might support the idea that VMT release and FTMH-closure are independent events, with the possibility of FTMH closure being dependent on VMT release, a hypothesis that was recently proposed and proven by Joondeph and coworkers^11^.

Other reports stratified baseline patient characteristics for FTMH eyes only but did not describe, if there was a significant impact of one or more baseline variables on treatment success, except FTMH diameter^4,8,12^. Recently Steel and coworkers analyzed various OCT-based parameters and their effect on FTMH closure after intravitreal ocriplasmin injections. They were able to show that the so called width factor, defined as the idiopathic macular hole base diameter (BD) minus the minimum linear diameter (MLD), amongst others, was the most predictive factor for FTMH closure. This is a major limitation of our current analysis as we had no access to the original OCT images and thus were not able to measure above mentioned parameters. However, this should be part of subsequent projects to further improve FTMH closure predictions in a multivariate approach^13^. The same applies to further anatomical parameters like central macular thickenss etc..

### Prediction of VMT release

We recently published a multivariate formula to calculate the individual probability for treatment success in eyes with VMT only^6^. We were able to show that the predicted chances match the clinical outcome of successful ocriplasmin treatments when using this formula. Such a formula could help patients as well as physicians to objectively decide whether or not to use ocriplasmin as a therapeutic approach. Our data show that a VMT resolution was achieved within 28 days in 63% of eyes treated with ocriplasmin. This is an interesting observation as FTMH closure was evident in only 38% of patients. Our results are in line with previous publications demonstrating a significantly higher rate of VMT resolution in eyes with VMT accompanied by a FTMH in comparison to eyes with VMT only. In the MIVI 6/7 trials, the observed differences were 50% versus 26.5%, respectively (OR = 2.1; CI 1.1–3.7)^14^. The reasons for these observations remain unclear.

The univariate analysis of predictors for VMT resolution in eyes with FTMH and VMT revealed that age and VMT diameter had a significant impact on treatment success, both of which were also shown to have the strongest effect on VMT resolution in a previous project in eyes with pure VMT^6^. Furthermore, the ORs herein were in comparable ranges with those in our previous publication^6^. Thus, we used our formerly published formula to predict the individual probability of VMT resolution in eyes with VMT only, to evaluate the forecast reliability of VMT release in eyes with FTMHs and VMT with matching results, concluding that VMT release can be predicted when using this formula.

Comparing the ORs herein with those of a meta-analysis done by Chatziralli et al.^4^ and results taken out of the EXPORT study^15^, some differences were obtained. Whereas in both publications being phakic was associated with treatment success, herein pseudophakic eyes had a higher chance for VMT release. We attribute these differences to the fact that in the INJECT study 100% of eyes were phakic as well as to the overall limited number of eyes included into the analysis. However, the overall impact of lens status on VMT release remains undetermined and might need further investigation, because the latter was recently demonstrated to be only a surrogate parameter for age^6,11^. Nevertheless, the effect power of all other variables is somewhat lower (lower ORs), despite being in the same range.

### Comparison of the predictability of VMT release and FTMH closure

The varying results and the different capability in the individual prognosis of ocriplasmin-induced treatment success between eyes with VMT only and those with FTMHs and VMT might be explained by different pathophysiological processes. Whereas VMT is considered to be a solely anterior-posteriorly directed traction on the fovea, which in turn is immediately affected by various patient characteristics like age or VMT size^16,17^, it is assumed that for FTMHs, additional tangentially oriented forces from persistent vitreous plaques or ERM formations play a crucial role in maintaining and enlarging the hole^18–20^. Taking our results into account one might speculate that VMT and FTMH with VMT are two different disease entities and the individual probability to predict treatment success between the latter varies. The prediction of ocriplasmin treatment success in eyes with pure VMT can be judged by using the previously published formula, whereas to date it seems not to be possible to estimate the treatment effect in a multivariate approach in eyes with FTMHs and VMT when analyzing known and established predictors^11^. However, there seems to be at least a potential interrelation between VMT release and FTMH closure as it was recently shown that FTMHs less than 250 µm were significantly associated with VMT release by day 28 and FTMH closure by month 6^11^.

As success rates of VMT release^3,5^ and FMTH closure^4^ still varies in the literature and a common consensus of ideal patients for intravitreal ocriplasmin is still missing^15^, there seems to be a continuing medical need for further improvement in patient selection to increase overall treatment success. The next step could be the use of artificial intelligence (AI) to analyze OCT scans^21^ to help ophthalmologists in the decision making process to find the best treatment option for each individual patient affected. In comparison to enzymatic vitreolysis using ocriplasmin with an overall success rate of FTMH closure of 38% herein, vitrectomy showed a success rate of more than 90%^22^. To the best of our knowledge the effect of age, gender, and lens status on treatment success were not evaluated so far. However, FTMH size and various surgical techniques, like ILM peeling, inverted flap procedure, and type of endotamponade etc.…) have shown to be beneficial^23^. Whereas the success rates are higher with vitrectomy, the latter still induces a more severe surgical trauma as compared to intravitreally applied ocriplasmin and complications can occur with both treatment modalities like RPE alterations, retinal detachments, cystoid macular edema, subretinal neovascular membranes, endophthalmitis, etc.^24,25^. This in turn supports our efforts to enhance patient selection for either therapeutical approach.

## Data Availability

The datasets used and analyzed during the current study are available from the corresponding author on reasonable request.

## References

[1] Stalmans P (2016). A retrospective cohort study in patients with tractional diseases of the vitreomacular interface (ReCoVit). Graefes. Arch. Clin. Exp. Ophthalmol..

[2] Parravano M (2015). Vitrectomy for idiopathic macular hole. Cochrane Database Syst. Rev..

[3] Stalmans P (2012). Enzymatic vitreolysis with ocriplasmin for vitreomacular traction and macular holes. N. Engl. J. Med..

[4] Chatziralli I (2016). Ocriplasmin use for vitreomacular traction and macular hole: A meta-analysis and comprehensive review on predictive factors for vitreous release and potential complications. Graefes. Arch. Clin. Exp. Ophthalmol..

[5] Maier M (2015). Ocriplasmin as a treatment option for symptomatic vitreomacular traction with and without macular hole: First clinical experiences. Ophthalmologe.

[6] Paul C (2018). Calculating the individual probability of successful ocriplasmin treatment in eyes with VMT syndrome: A multivariable prediction model from the EXPORT study. Br. J. Ophthalmol..

[7] Khanani AM (2019). Ocriplasmin treatment leads to symptomatic vitreomacular adhesion/vitreomacular traction resolution in the real-world setting: The phase IV ORBIT study. Ophthalmol. Retina.

[8] Steel DHW (2021). Ocriplasmin for vitreomacular traction in clinical practice: The INJECT study. Retina.

[9] Duker JS (2013). The international vitreomacular traction study group classification of vitreomacular adhesion, traction, and macular hole. Ophthalmology.

[10] Haller JA (2015). Efficacy of intravitreal ocriplasmin for treatment of vitreomacular adhesion: Subgroup analyses from two randomized trials. Ophthalmology.

[11] Joondeph BC (2021). Prognostic factors associated with ocriplasmin efficacy for the treatment of symptomatic vitreomacular adhesion and full-thickness macular hole: Analysis from four studies. J. Ophthalmic Vis. Res..

[12] Tadayoni R (2019). Assessment of anatomical and functional outcomes with ocriplasmin treatment in patients with vitreomacular traction with or without macular holes: Results of OVIID-1 trial. Retina.

[13] Steel DH (2016). Predicting macular hole closure with ocriplasmin based on spectral domain optical coherence tomography. Eye (London).

[14] Jackson TL (2016). Baseline predictors of vitreomacular adhesion/traction resolution following an intravitreal injection of ocriplasmin. Ophthalmic Surg. Lasers Imaging Retina.

[15] Bertelmann T (2017). The predictability of ocriplasmin treatment effects: is there consensus among retinal experts? Results from the EXPORT study. Graefes Arch. Clin. Exp. Ophthalmol..

[16] de Smet MD, Gad Elkareem AM, Zwinderman AH (2013). The vitreous, the retinal interface in ocular health and disease. Ophthalmologica.

[17] Bertelmann T, Witteborn M, Mennel S (2012). Pseudophakic cystoid macular oedema. Klin. Monbl. Augenheilkd..

[18] Spaide RF (2005). Macular hole hypotheses. Am. J. Ophthalmol..

[19] Madi HA, Masri I, Steel DH (2016). Optimal management of idiopathic macular holes. Clin. Ophthalmol..

[20] Chen Q, Liu ZX (2019). Idiopathic macular hole: A comprehensive review of its pathogenesis and of advanced studies on metamorphopsia. J. Ophthalmol..

[21] Arrigo A (2020). Vitreomacular traction quantitative cutoffs for the assessment of resolution after ocriplasmin intravitreal treatment. Sci. Rep..

[22] Ittarat M (2020). Literature review of surgical treatment in idiopathic full-thickness macular hole. Clin. Ophthalmol..

[23] Steel DH, Lotery AJ (2013). Idiopathic vitreomacular traction and macular hole: A comprehensive review of pathophysiology, diagnosis, and treatment. Eye (London).

[24] Javid CG, Lou PL (2000). Complications of macular hole surgery. Int. Ophthalmol. Clin..

[25] Stalmans P (2010). Intravitreal injection of microplasmin for treatment of vitreomacular adhesion: Results of a prospective, randomized, sham-controlled phase II trial (the MIVI-IIT trial). Retina.

